# 2458 Determining the prevalence and associated multilevel characteristics of undiagnosed diabetic retinopathy

**DOI:** 10.1017/cts.2018.247

**Published:** 2018-11-21

**Authors:** Kristen Nwanyanwu, Marcella Nunez-Smith, Mayur Desai, Thomas Gardner

**Affiliations:** Yale School of Medicine

## Abstract

OBJECTIVES/SPECIFIC AIMS: Diabetic retinopathy is the leading cause of blindness in adults aged 25–64 years. The prevalence of diabetic retinopathy is projected to increase 4-fold by 2050. Racial and ethnic minorities have a higher prevalence and greater severity of diabetic retinopathy. Over 50% of racial and ethnic minorities are not screened for diabetic retinopathy per guidelines. With timely diagnosis and sight-saving treatment, blindness from diabetic retinopathy is largely preventable. The objective of this study is to identify racial and ethnic disparities in the population that do not know they have diabetic retinopathy and to compare those disparities to those in the population that do know they have diabetic retinopathy. METHODS/STUDY POPULATION: Specifically, we have identified a nationally representative survey and clinical examination data to estimate the prevalence of undiagnosed diabetic retinopathy, to identify racial and ethnic disparities in that population, and to compare those disparities in the population with known diabetic retinopathy. We hypothesize that racial and ethnic disparities will be higher in the population with undiagnosed diabetic retinopathy in comparison to the population with known diabetic retinopathy. RESULTS/ANTICIPATED RESULTS: We hypothesize that racial and ethnic disparities will be higher in the population with undiagnosed diabetic retinopathy in comparison to the population with known diabetic retinopathy. The results of that analysis will instruct qualitative interviews that will advance the understanding of the factors that contribute to the decision whether to be screened for diabetic retinopathy. A decision tree will be created to categorize the hierarchy of barriers and facilitators. DISCUSSION/SIGNIFICANCE OF IMPACT: A better understanding of the population with undiagnosed diabetic retinopathy and the factors that influence the decision to get screened will help us not only to address disparities in diabetic retinopathy, but also to prevent blindness from retinopathy.

